# Yttrium-induced tunable bandgap for optical data storage applications

**DOI:** 10.1039/d4ra05458j

**Published:** 2024-09-19

**Authors:** Surbhi Agarwal, D. K. Dwivedi, Pooja Lohia, Manoj Kumar Gupta

**Affiliations:** a Photonics and Photovoltaic Research Lab, Department of Physics and Material Science, Madan Mohan Malviya University of Technology Gorakhpur 273010 India todkdwivedi@gmail.com; b Department of Electronics and Communication Engineering, Madan Mohan Malviya University of Technology Gorakhpur 273010 India; c CSIR – Advanced Materials and Processes Research Institute Madhya Pradesh 462026 India; d Academy of Scientific and Innovative Research (AcSIR) Ghaziabad 201002 India

## Abstract

Phase-change memory (PCM) relies on the characteristics of phase-change materials that exhibit slow resistance state changes and enable multilevel operation with minimal resistance drift. They are emerging as promising candidates for artificial intelligence applications inspired by neuroscience and require high volumes of data. However, achieving the necessary qualities, such as thermal stability and fast operation speed, simultaneously is still a major obstacle for PCM materials. The present study investigated the linear and nonlinear optical and electronic properties of Te_(1−*x*)_(GeSe_0.5_)Y_*x*_ (*x* = 0.05, 0.1, 0.15) thin films deposited *via* a thermal evaporation technique by structural characterization (using XRD), surface morphology analysis (using SEM), and elemental composition analysis (using EDX). Transmission spectra ranging from 500 to 2500 nm were obtained using a UV-visible spectrophotometer to determine the optical properties. The refractive index (*n*) and extinction coefficient (*k*) were also determined, and Tauc's relationship was applied to assess the optical absorption data. The absorption coefficient (*α*) was determined utilizing the Urbach relation. The Wemple–DiDomenico model was employed to calculate the nonlinear refractive index. Furthermore, the dielectric properties, loss tangent, and surface/volume energy loss functions were determined. The optical energy bandgap of the thin films revealed the allowed indirect transitions. The observed enhancement of the optical parameters suggested that the investigated composition is appropriate for different photonic applications.

## Abstract 2

Linear and nonlinear optical and electronic properties of Te_(1−*x*)_(GeSe_0.5_)Y_*x*_ thin films have been studied. Transmission spectra ranging from 500 to 2500 nm were obtained using a UV-visible spectrophotometer to calculate optical properties.

## Graphical abstract

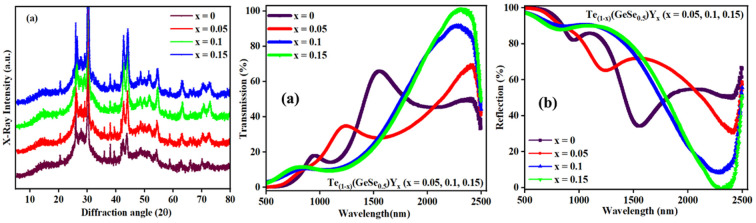

## 1. Introduction

In recent years, there has been an exponential growth in the demand for mobile electronics, data storage and processing in integrated circuits,^1^ quantum computing, blockchain, and artificial intelligence. Chalcogenide materials are alloys containing at least one of the elements sulfur (S), selenium (Se), or tellurium (Te). These elements serve as the fundamental building blocks for revolutionary concepts in microelectronics and microtechnologies. Chalcogenide materials play a pivotal role in advancing many fields, such as infrared optics, spintronics, thermoelectricity, and the design of non-volatile and neuromorphic memory components. Some examples of these components include PCM (phase-change memory), NLO (nonlinear optics), OTS (Ovonic threshold switching devices), TE (thermoelectrics), and FESO (ferroelectric spin–orbit devices). There is also a new class of crystals that have phase-matching conditions in the whole transparency regime, called full-wavelength phase-matching crystals. Borates^2^ show increasing potential to form noncentrosymmetric structures, possess wide optical transparency ranges, maintain excellent chemical stability, and exhibit significant polarizabilities. These characteristics enable them to achieve a combination of suitable second-order nonlinear optical (NLO) coefficients and birefringence, making borates a distinctive and exceptional class of materials for optical applications. Guanidinium tetrafluoroborate^3^ (C(NH_2_)_3_BF_4_ or GFB) has been experimentally shown to produce frequency-doubled light near its cut-off wavelength.

In the 1960s, Ovshinsky^4–6^ made groundbreaking discoveries in resistive switching and phase-change memory effects utilizing chalcogenides, sparking extensive research into amorphous semiconductors. His discoveries ignited global interest and paved the way for the commercialization of digital RAM disks. Furthermore, they spurred the development of innovative phase-change materials, driving advancements in device fabrication techniques. In 1970s and 1980s, Ovshinsky^4,5^ with the help of many other researchers^7,8^ founded a company for producing amorphous semiconductors at the industrial level. Various engineering materials were developed by the company for the fabrication of commercialized memory devices. Regrettably, the physical limit has now been reached in traditional memory systems. Therefore, there is an urgent need for a new memory alternative that offers high thermal stability, low power consumption, and enhanced storage capacity. Solid-state drives (SSDs) incorporating dynamic random-access memories (DRAMs) and NAND Flash are in competition due to the growing demand for faster, denser, and more reliable storage solutions, juxtaposed with the limitations of DRAMs (volatile nature, increased cost, and power consumption) and NAND (poor reliability and slow read and write speeds).^9,10^

Alloys consisting of the chalcogenide elements selenium (Se) and tellurium (Te) and intrinsic semiconductor element germanium (Ge) offer exceptional electronic, optical, magnetic, and thermal properties.^11–13^ Selenium and tellurium are commonly employed chalcogens with potential applications in various electronic devices. However, synthesizing them in their pure form on a large scale is not practical. Telluride-based glassy alloys, while offering extensive infrared transmission and optical phase recording capabilities, exhibit a low glass-forming ability. In a similar manner, pure selenium, although it does form a good glass, it has a limited lifespan and also lacks sensitivity. It has been observed that alloys of selenium and tellurium demonstrate improved properties compared to their pure forms, including enhanced hardness, aging resistance, crystallization, and transmittance. Selenium–tellurium (Se–Te) alloys have attracted attention for their widespread industrial and scientific significance. Nevertheless, these alloys face certain challenges, such as a limited reversibility, relatively low crystallization temperature, and susceptibility to aging effects.

To overcome these shortcomings, researchers have explored the enhancement of the low thermo-mechanical properties of Se–Te glassy alloys through the introduction of other metalloids as suitable impurities. This approach results in the synthesis of multi-component composites, expanding the potential applications of Se–Te alloys and rendering them as multifunctional. Incorporating germanium (Ge) into the Se–Te^14,15^ matrix effectively addresses the limitations observed in the binary Se–Te alloy. The introduction of Ge involves cross-linking with the Se-chain, thereby modifying the bonds and strengthening the overall bond structure within the system. Furthermore, the introduction of germanium contributes to an expanded glass formation region. The compatibility in the size and electronegativity among the elements in the Ge–Se–Te matrix results in the creation of high-quality, stable melts. Earlier study conducted by one of the researchers thoroughly investigated the thermal, optical, and electrical properties of both the amorphous Ge–Se–Te bulk system and its thin films. Ge–Te–Se ternary alloys have great advantages due to their high photosensitivity, greater crystallization temperature, and slow aging effects. In the present work, yttrium was substituted with respect to tellurium in the host matrix of Ge–Te–Se. Yttrium increases the electrical resistivity of the crystalline state and also the thermal stability of the amorphous phase. The introduction of a small quantity of impurity allows for the customization of the alloy properties to align with industry requirements. The thermal properties were investigated earlier using DSC measurements.^16^ This research also indicated that the incorporation of yttrium (Y) into the Ge–Te–Se matrix enhances the thermal properties, glass stability, and appears to improve the optical storage^17,18^ process. Glassy alloys based on Ge–Te–Se exhibit a rapid transition between crystallization and amorphization. The incorporation of Y into Se-based glassy alloys results in an expansion of the glassy region, which affects the reliability and accuracy of data processing in phase-change memory.

The primary objective of the work is to analyze (i) the type of transition, (ii) bandgap variation, (iii) variation in the linear and nonlinear optical characteristics, and (iv) morphological and compositional changes. XRD confirmed the synthesized material was amorphous in nature due to the absence of sharp peaks, in agreement with the Raman analysis. Surface morphology imaging was done by scanning electron microscopy (SEM). An energy dispersive X-ray (EDX) study was also performed to verify the composition of the materials in the deposited films. The linear and nonlinear optical parameters were determined by UV-visible spectroscopy.

## 2. Experimental

### 2.1. Thin-film preparation

Quaternary TGSY (Te_(1−*x*)_(GeSe_0.5_)Y_*x*_) glassy alloys with varying *x* values (0.05, 0.1, 0.15) were synthesized through the melt-quench technique. High-purity elemental precursors (Te, Ge, Se, and Y), each with a purity of 5 N, were proportionally weighed based on their atomic percentages. These materials were then placed in a silica tube measuring 5 cm in length and 8 mm in diameter. The tube was sealed and evacuated to 10^−5^ Pa. Subsequently, the sealed ampoules underwent a carefully controlled heating process in a furnace, with the temperature gradually increasing at a rate of 3–4 °C per minute. To maintain consistency, the ampoules were gently rocked during the heating process, achieved by periodically rotating a ceramic rod inside each ampoule. The process required 11 to 12 h of rocking to reach 1000 °C. The resulting molten substance was rapidly quenched by quick cooling in a container with ice water. The cooled substance was taken out by breaking open the ampoule and ground into powder using a mortar and pestle. Additionally, thin films, approximately ∼500 nm in thickness, were prepared through the thermal evaporation method.

### 2.2. Characterization

**Fig. 1:**
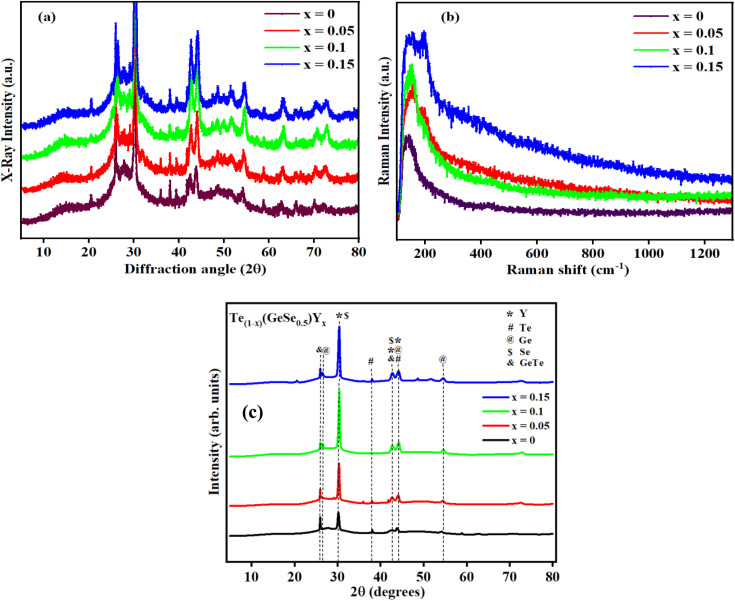
(a) X-ray diffractograms, (b) Raman spectra, (c) Rietveld refined XRD of Te_(1−*x*)_(GeSe_0.5_)Y_*x*_ (*x* = 0, 0.05, 0.1, 0.15) thin-film samples.

**Fig. 2:**
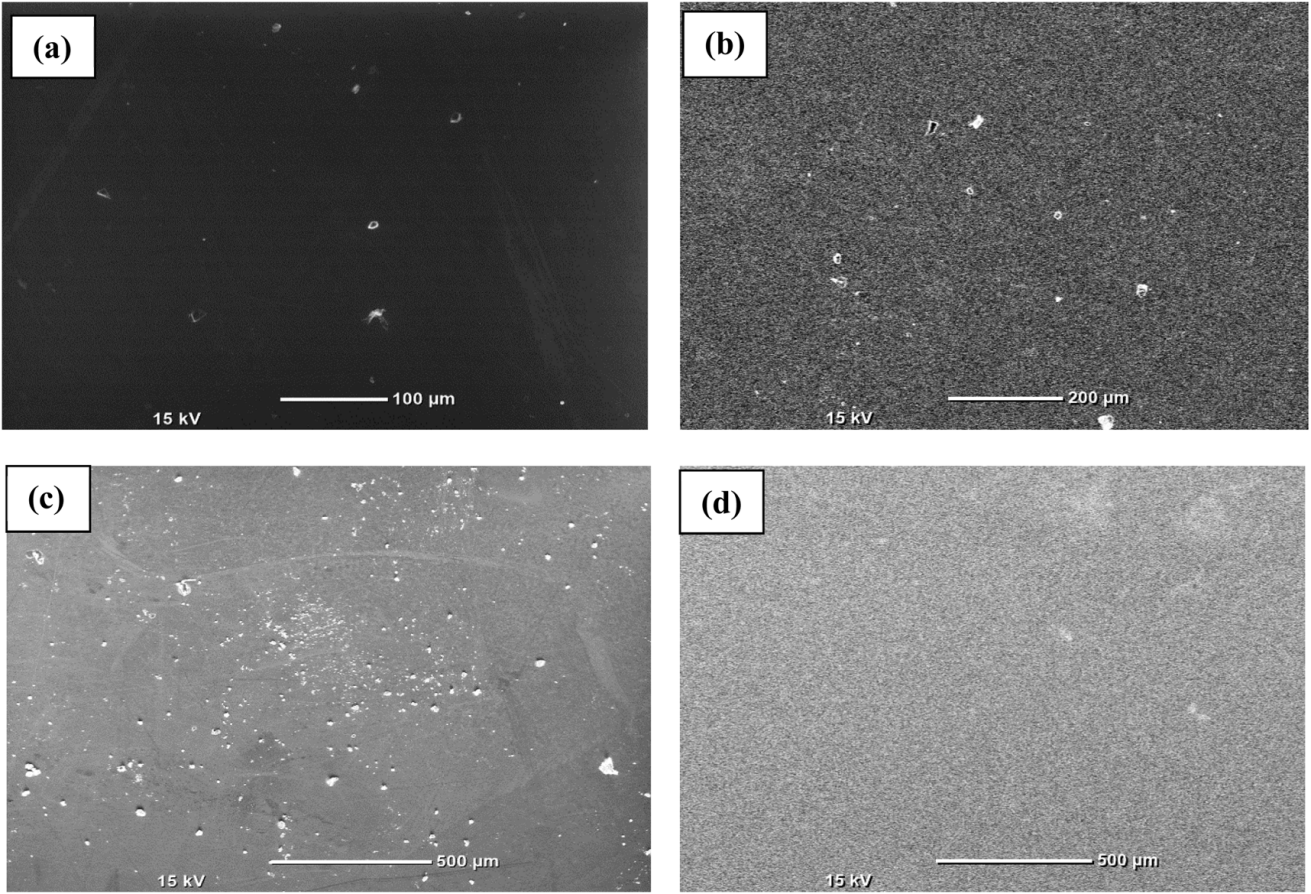
SEM images for surface morphological study of the Te_(1−*x*)_(GeSe_0.5_)Y_*x*_ films. (a) *x* = 0, (b) *x* = 0.05, (c) *x* = 0.1, (d) *x* = 0.15.

**Fig. 3:**
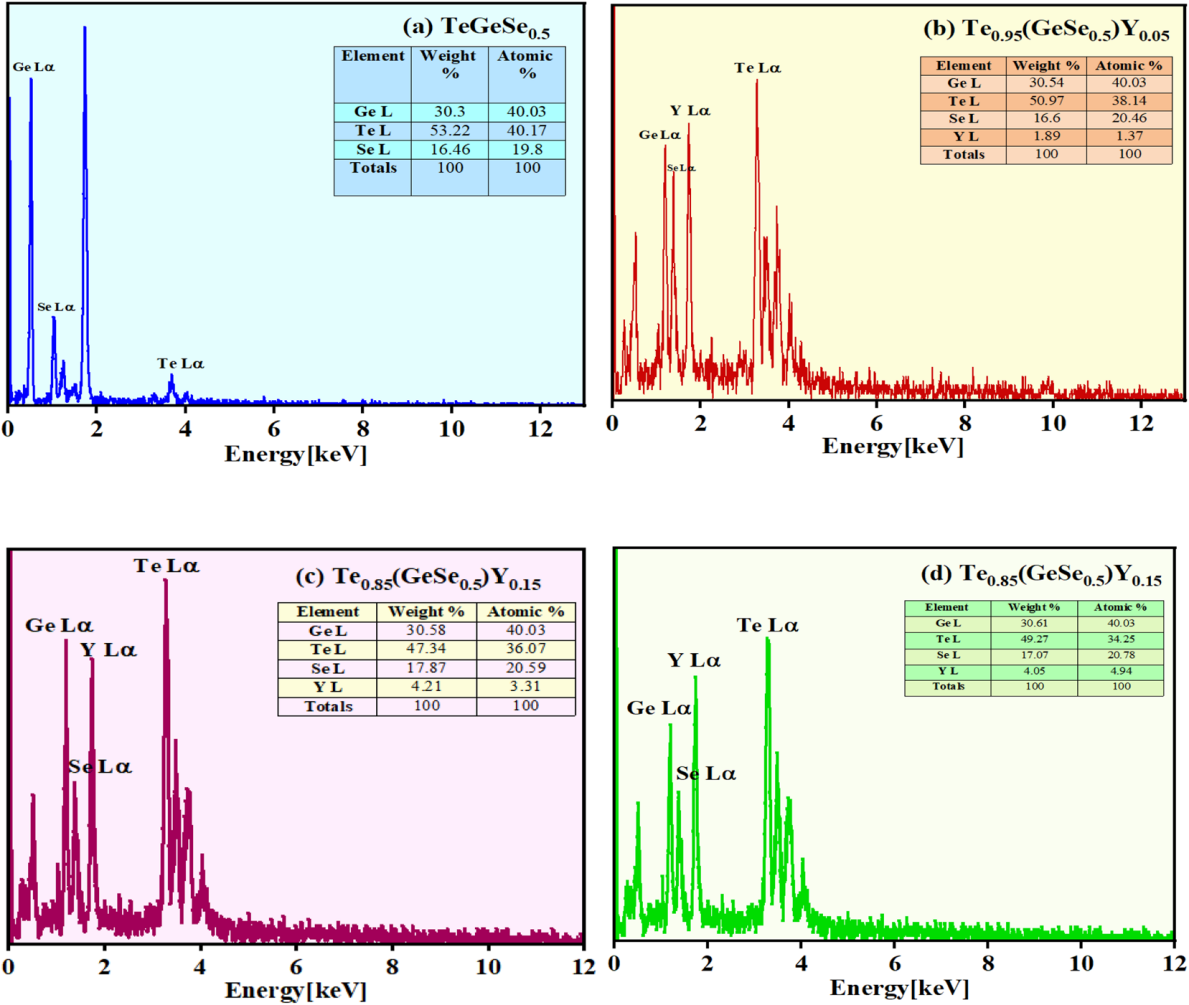
EDX spectra for the compositional study of the Te_(1−*x*)_(GeSe_0.5_)Y_*x*_ thin films (a) *x* = 0, (b) *x* = 0.05, (c) *x* = 0.1, (d) *x* = 0.15.

First, every sample underwent X-ray diffraction (XRD) analysis with Cu–K (1.54) radiation within a 10° to 80° range of scanning using a Rigaku Smart Lab 9 kW instrument. The amorphous nature of the observed samples was assured by the absence of a distinct peak, as illustrated in Fig. 1(a). Some minor peaks with low intensities and broad humps, namely, the first sharp diffraction peak (FSDP) and second sharp diffraction peak (SSDP), were observed, indicating the characteristics of intermediate range-ordering in the parent alloy. The material's morphology was determined through field-emission scanning electron microscopy (SEM) utilizing MA15/18 and 51N1000 instruments. The homogeneity of film deposition was validated by the SEM images presented in Fig. 2. An EDX plot is presented in Fig. 3.

Raman spectroscopy, as depicted in Fig. 1(b), was employed to study the chemical formation, crystallinity, phase, and molecular interactions. Optical absorption spectra were recorded using a double-beam UV-visible spectrophotometer (Jasco V-770, FLH-741) within a 500–2500 nm wavelength range. In addition, IR transmission spectra were obtained utilizing an FTIR spectrophotometer (Shimadzu IRSpirit QATR-S FTIR spectrometer) in the range of 400 to 4000 per cm, with a resolution of 2 per cm. The linear and nonlinear optical characteristics were evaluated from the transmission spectra utilizing a variety of relationships.

## 3. Results and discussion

### 3.1. Structural analysis using XRD and Raman spectroscopy

Fig. 1(a) presents the XRD analysis for the Te_(1−*x*)_(GeSe_0.5_)Y_*x*_ system with *x* values of 0, 0.05, 0.1, and 0.15. The diffraction angle 2θ was scanned from 10° to 80°. The XRD pattern of bulk Te_(1−*x*)_(GeSe_0.5_)Y_*x*_, as depicted in Fig. 1(c), illustrated the impact of Y substitution for Te. Initially, for GeTeSe_0.5_ (*x* = 0), the diffraction peaks precisely aligned with the standard pattern of ICSD23, indicative of a rhombohedral structure with minor traces of phase-segregated elemental Te. However, the effect of Y substitution became apparent through the broadening of the peak width and shift of the peak positions toward higher 2-theta values. The widening of the peaks indicated the increased Y content causing lattice distortion. The shift toward higher 2-theta values resulted from the compression of the lattice dimensions upon substituting the smaller Y atom for Te. This describes the analysis of the Se-substituted samples using Rietveld refinement with GeTeSe_0.5_ as a parent structure. The calculated and observed refinement patterns aligned well with the experimental measurements, indicating good agreement. The results, summarized in Table 1, reveal a systematic decrease in the lattice parameters, crystallite size, and volume for the Te_(1−*x*)_(GeSe_0.5_)Y_*x*_ samples. The broadening of the peak full width at half maximum (FWHM) suggested a reduction in the coherent scattering domains, likely attributed to local distortion caused by Y's smaller atomic size within the GeTeSe_0.5_ structure.

**Table 1:** Rietveld refinement data of the Te_(1−*x*)_(GeSe_0.5_)Y_*x*_ glasses

Chemical formula	GeTeSe_0.5_	*x* = 0.05	*x* = 0.1	*x* = 0.15
Crystal system	Rhombohedral	Rhombohedral	Rhombohedral	Rhombohedral
Space group	*R*3*m*:H	*R*3*m*:H	*R*3*m*:H	R3*m*:H

**Unit cell parameters** Å				
a	4.16872	4.14658	4.11887	4.07314
b	4.16872	4.14658	4.11887	4.07314
c	10.4781	10.3781	10.1973	10.2481
c/a	2.5135	2.5028	2.4757	2.5160
Unit cell volume Å^3^	160.97420	158.72481	156.11487	155.37860

***R* values**				
*R*_wp_	0.05674	0.06654	0.07167	0.03349
*RF*_2_	0.06487	0.06741	0.08483	0.02471
*X* ^2^	1.997	1.8950	2.017	1.648
Crystallite size (nm)	32.7	30.9	28.6	27.9

Raman spectroscopy, as shown in Fig. 1(b), was conducted to investigate the chemical structure, phase, crystallinity, and molecular interactions of the samples. The vibrational mode observed at ∼160 cm^−1^ corresponded to the Ge–Te rhombohedral structure. It could be observed from all the spectra that there were negligible changes in the amorphous phase following Y doping.

### 3.2. Surface morphology study using SEM-EDX

Fig. 2 shows the SEM images of the thin films, showing the homogeneous deposition of the material on the substrate, indicating there was no cluster formation. Fig. 3 presents the EDX spectrum of the Te_(1−*x*)_(GeSe_0.5_)Y_*x*_ system. The spectrum displayed maxima for the Ge, Te, Se, and Y elements, and their ratios were close to those of the nominal sample composition. Using *k*-factors for each element, it was estimated that the atomic percentage ratios in the analyzed alloy were approximately 40% Ge, 38% Te, 20% Se, and 2% Y. The error in estimating these elements did not exceed 2%.

### 3.3. Study of the optical properties

Many optical features of semiconducting materials depend on understanding the optical absorption of the electromagnetic wave spectra, which is important fundamental knowledge. The extent of absorption of films may be impacted by numerous factors, including the composition of the film, doping components, doping ratios, and sample surface morphology. Thus, examination of the optical absorption and associated factors, for example, skin depth, optical density, extinction coefficient, and absorption coefficient, provides significant information. These parameters provide useful details regarding the characteristics and modifications of the electronics of the materials examined. For instance, it would be noteworthy to notice that the semiconductor material optical absorbance spectra show a sudden increase in specific photon energy. This increase might be related to electron changes that occur in the VB to CB.

It should be mentioned that momentum and energy are conserved throughout the absorption process. At the semiconducting material's absorption edge, both direct and indirect transitions occur. The electrons in the VB undergo transit to the CB as a result of these transitions, which are caused by the interactions between electromagnetic waves and the electrons. In general, simultaneous interactions with the lattice vibrations are involved in the indirect transitions. As a result, in optical transitions, the electron wave vector may change.^19,20^ Therefore, in order to determine the transition type and the energy value of the optical bandgap of the current chalcogenide Te_(1−*x*)_(GeSe_0.5_)Y_*x*_ (*x* = 0.05, 0.1, 0.15) thin-film samples, the research of optical absorption and absorption coefficient ‘*α*’ is given priority in the present investigation.

#### 3.3.1. Transmittance, reflectance, extinction coefficient, optical density, and skin depth

**Fig. 4:**
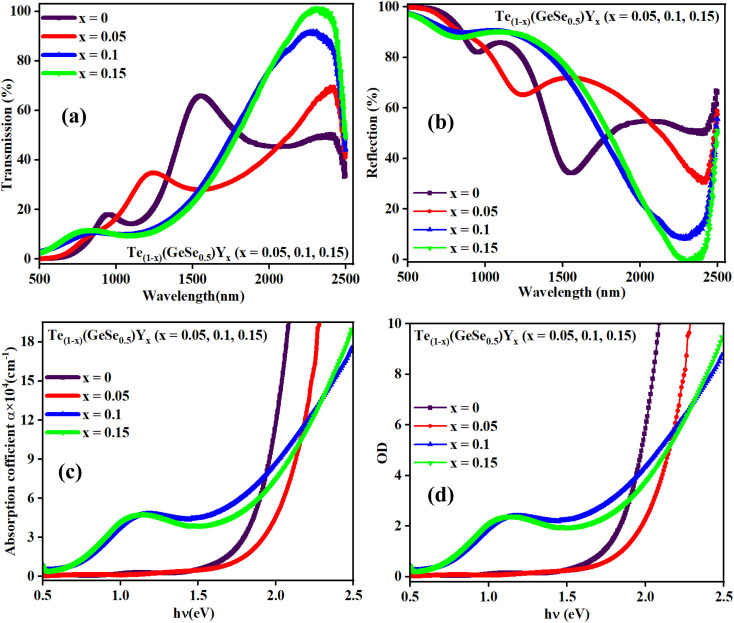
Variation in the (a) transmittance, (b) reflectance with wavelength, (c) absorption coefficient, and (d) optical density with the energy of the studied samples.

There was an increase in transmission with the wavelength for the Te_(1−*x*)_(GeSe_0.5_)Y_*x*_ (*x* = 0.05, 0.1, 0.15) thin films (Fig. 4(a)). The transmittance value was increased from 65% to 100% at 2300 nm wavelength. The consistent alignment of the wavelength positions between the extremes of the reflectance spectra (Fig. 4(b)) and the minima of the transmittance spectra, and *vice versa*, indicated the optical homogeneity of the thin films. At higher wavelengths, there was no absorption in the films. With the increase in Y content, the short-wavelength cut-off edge was shifted to longer wavelength, which means there was a red-shift. Meanwhile, the absorption cut-off edge was shifted to lower wavelength, which indicates there was a blue-shift. The transparency was notably higher in the infrared region, making these films potentially suitable for systems that utilize infrared, such as fiber optics. The noticed interference pattern in the film arose from the interference between the film surface and substrate.

Optical absorbance serves as a useful indicator offering insights into the bandgap and band structure of both amorphous and crystalline materials. The optical absorption edge arises from electronic transitions in the semiconductor. In this context, the region of the absorption edge fell within the scale of 500–1000 nm, and the absorption coefficient (*α*) was evaluated from the absorbance value utilizing the below equation:1
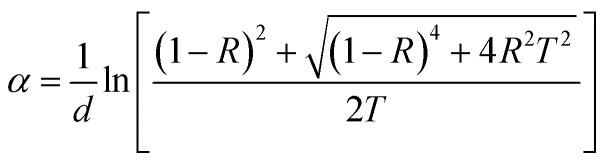
where *d* is the thickness of the film, and *T* and *R* denote the transmittance and reflectance of the material. The above equation can be simplified to:2
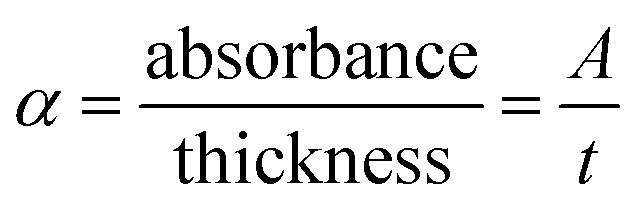
where *A* denotes the absorbance and *t* signifies the thickness of the deposited film. The absorption coefficient quantifies the rate at which the intensity of electromagnetic radiation decreases while traversing through the medium of a material. A higher absorption coefficient corresponds to an increased absorption of light by the material. The variation in ‘*α*’ with energy (shown in Fig. 4(c)) is a key factor in understanding how the material interacts with different wavelengths of light. The values of *α* here were in the order of 10^4^ cm^−1^, which increased with the increasing Y content. The existence of these absorption edges makes these materials promising candidates for optical-storage applications.^21^

The absorption capacity of the film is also indicated by another optical measure known as the optical density (OD). This indicates the extent to which a medium slows down transmitted light rays. The optical density is contingent upon the material concentration. It is associated with a subtle inclination of the atoms within the material to retain the absorbed energy from electromagnetic waves through vibrating electrons. This absorbed energy is subsequently re-emitted as a new disturbance for the propagating waves. Additionally, the dispersion of thin films, as indicated by their optical density, varies with thickness. Consequently, the velocity of electromagnetic waves is influenced by the optical density of the film material. As the optical density increases, the hindrance to the propagation of electromagnetic waves within the material intensifies. Fig. 4(d) illustrates the variation in OD (= *α* × *t*, where ‘*t*’ represents the film thickness and ‘*α*’ denotes the absorption coefficient), which increased with the Y incorporation. In thin films, the OD arises from the refraction and scattering of light, reflecting the speed of light within the material. The absorption is minimal (below 2%) at lower frequencies, leading to an improvement in electromagnetic wave transmission within a low-absorption range. This characteristic contributed to the high infrared (IR) transmission observed in Ge–Te–Se–Y chalcogenide thin films. Here, the optical density or the absorbance increased at higher energy values and it also increased with the Y content. For *x* = 0, the OD increased from 1.56 eV, to 1.63 for *x* = 0.05, 1.16 eV for *x* = 0.1, and 1.07 eV for *x* = 0.15. The optical density increased for *x* = 0.1 and 0.15 and it increased at lower values of energy compared to for *x* = 0, 0.05. The higher optical density was due to the bonding contribution of yttrium, where the bonding ratio Ge–Te/Ge–Y decreased with the Y content in the Ge–Te–Se–Y alloys. This behavior has been observed in other researchers' work also.^22–24^

**Fig. 5:**
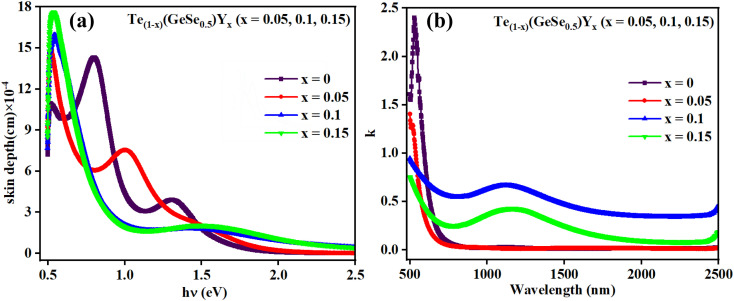
(a) Variation of skin depth ‘*δ*’ spectra with the photon energy, (b) dependence of the k-spectra with the wavelength for the Te_(1−*x*)_(GeSe_0.5_)Y_*x*_ thin-film samples.

In the spectral region with high absorbance, incident photon energy is absorbed by the semiconducting film. The penetration depth, also known as the skin depth (*δ*), is defined as the thickness of the film at which the optical density of the incident photon reaches 1/e of its value at the surface of the film. This depth of penetration is influenced by various parameters, including the film conductivity, material density of the film, and incident photon frequency, as well as the morphology of the film surface and its microstructure. The electrical and optical conductivities of semiconductors are contingent upon the optical bandgap energy and electronic transitions. Hence, there exists a correlation between the penetration depth (*δ*) of semiconductor films and their optical properties. The penetration depth or skin depth (*δ*) is related to the coefficient of absorption (*α*) through the equation:^25,26^*δ* (cm) = 1/*α*. The relationship between the penetration depth (*δ*) and the photon energy incident on the Te_(1−*x*)_(GeSe_0.5_)Y_*x*_ thin-film surface is depicted in Fig. 5(a).

It was observed that the penetration depth (*δ*) diminished with the incorporation of the Y content. This reduction in *δ* was correlated with an escalation in the absorbance of the film. Furthermore, during synthesizing, it was noted that the darkness of the films intensified with the increasing Y content. Additionally, the penetration depth decreased with the increase in the energy of the incident photons up to a certain value. The value of energy at which *d* becomes zero, observed across all the film samples, is termed the cut-off energy (*E*_cutoff_), approximately amounting to 2 eV for the films under examination. The corresponding wavelength at this energy level is denoted as the cut-off wavelength (*λ*_cutoff_), nearly equaling 623 nm for the current samples. Additionally, for photon energies exceeding the *E*_cutoff_ value (>2 eV), the penetration depth diminished (*δ* = 0), indicating the films' highest absorbance. Conversely, the propagated electromagnetic waves amplitude decreased at lower energies. Subsequently, the penetration depth (*δ*) demonstrated an increment toward the lower energy side, highlighting its strong dependence on the transmittance and reflectance values. This behavior aligns with previous findings reported in other work for similar semiconducting films.^27,28^

The rate at which electromagnetic waves travel through a material is dictated by the complex refractive index, typically represented as ñ = *n* − *ik*, where *k* stands for an optical parameter known as the coefficient of extinction, attenuation coefficient, or absorption index, while *n* indicates the refractive index. This optical constant, *k*, characterizes the reduction in the amplitude of incident electric-field oscillations. Consequently, the optical characteristics of the film material are influenced by the interaction between the electric-field component of the incident electromagnetic waves and the atoms of the material. Therefore, *k* plays a critical and foundational role in understanding the propagation of light waves through any material, as its value determines the extent of energy dissipation or loss due to absorption. The coefficient of extinction, *k*, can be expressed as *k*= (*αλ*/4π), where *α* represents the absorption coefficient and *λ* denotes the wavelength. Fig. 5(b) describes the spectral variation of this coefficient concerning the wavelength of incident photons. It displays there was a significant decrement in *k* values for the Te_(1−*x*)_(GeSe_0.5_)Y_*x*_ films as the wavelength of the incident photons increased in the NIR region. The refractive index, extinction coefficient, and nonlinear parameters are all interconnected with the absorption coefficient. Its investigation holds significance primarily for estimating the propagation patterns of electromagnetic waves within the examined thin films with minimal damping or scattering. The *k* value explains the scattering, attenuation, or absorption of incident electromagnetic waves. Notably, the calculated lower *k* values and high transmission window render these materials highly suitable for near-infrared (NIR) and infrared (IR) optical applications. A similar behavior was observed by Mott and Davis^29^ in various other amorphous semiconductor materials. Additionally, the *k* value plays a vital role in dielectric studies.

#### 3.3.2. Optical bandgap, Tauc parameter, and Urbach energy

**Fig. 6:**
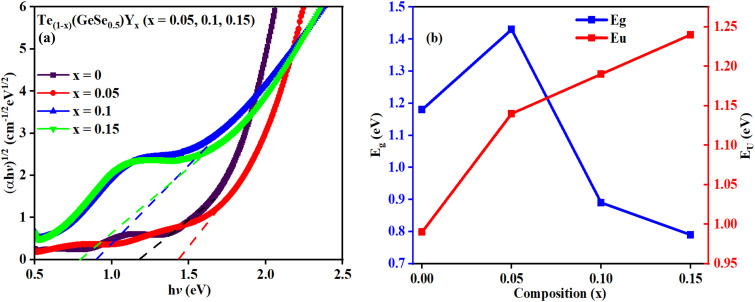
(a) Evaluation of the indirect bandgaps of the studied films, (b) *E*_g_ and *E*_U_ relationship.

The optical absorption mechanism at the fundamental edge is elucidated through the concept of band-to-band transitions. Absorption takes place when photons possess energy equal to or greater than the band gap. The absorption coefficient, representing the likelihood of electronic transitions across the forbidden gap, quantifies the number of absorbed photons per incident photon. A material's optical bandgap can be inferred from the absorption coefficient. Utilizing this coefficient, photon energies can be approximated to match the bandgap energies of the chalcogenides. Three distinct regions are observed: In the initial region, characterized by weak absorption (*α* < 1 cm^−1^), the absorption tendencies are influenced by the synthesis methods, material purity, and temperature history; The second region (10 < *α* < 10^4^ cm^−1^) correlates to the Urbach region, where absorption happens between localized and extended states; The third region exhibits a higher absorption coefficient (*α* > 10^4^ cm^−1^), known as the Tauc region.^30–32^ The optical bandgap, influenced by structural randomness, can be computed using the Tauc equation:

3 *αhv* = *P*(*hv* − *E*_g_)^*m*^

where ‘*P*’ represents the Tauc parameter, while ‘*m*’ denotes the exponent. The exponent ‘*m*’ determines the nature of the electronic transition within the band gap. Different values of ‘*m*’ correspond to distinct types of transitions: for instance, *m* = 2 indicates an indirect allowed transition, 1/2 represents a direct allowed transition, 3 signifies an indirect forbidden transition, and 3/2 indicates a direct forbidden transition. The analysis of the studied thin-film absorption data suggested a fit for *m* = 2, indicating an indirect allowed transition type. Fig. 6 illustrates the plot of (*αhυ*)^1/2^*versus hυ*, confirming the presence of an indirect allowed transition. The linear portion of the plot, intersecting the *x*-axis, provides the value of the optical bandgap (*E*_g_), as detailed in Table 2.

**Table 2:** Variation in the optical properties of the Te_(1−*x*)_(GeSe_0.5_)Y_*x*_ (*x* = 0.05, 0.1, 0.15) thin films

Optical parameters	Te_(1−*x*)_(GeSe_0.5_)Y_*x*_			
	*x* = 0	*x* = 0.05	*x* = 0.1	*x* = 0.15
*E*_g_ (eV)	1.18	1.43	0.89	0.79
*P* ^1/2^ (cm^−1/2^ eV^−1/2^)	165.87	158.61	151.37	144.28
*E*_U_ (eV)	0.99	1.14	1.19	1.24
*σ* × 10^−2^ (steepness parameter)	18.45	17.02	16.39	15.14
*S*_e−p_	3.62	3.91	4.26	4.61
*E*_d_ (eV)	21.57	23.31	25.84	28.69
*E*_o_ (eV)	1.34	1.32	1.29	1.27
*f* = *E*_o_*E*_d_	28.90	30.76	33.33	36.43
*M*_−1_	16.09	17.65	20.03	22.59
*M*_−3_	8.96	10.12	12.03	14.01
*n*_o_	3.89	3.91	3.95	3.98
*ε*_∞_	23.23	26.65	28.03	30.59
*λ*_o_ (nm)	642.71	684.09	734.28	788.35
*s*_o_ (nm^2^ × 10^−8^)	10.25	11.47	13.89	15.34
*ε*_*L*_	3.67	5.84	6.15	8.79
*N*/m* (× 10^56^) (m^−3^ kg^−1^)	0.1556	0.1570	0.1684	0.1725
Plasma frequency *ω*_P_ (× 10^14^)	3.98	3.57	3.24	3.07
*η*_opt_	1.60	1.66	1.73	1.81
*χ* ^1^ (esu)	1.1297	1.1314	1.1357	1.1387
*χ* ^3^ × 10^−11^ (esu)	2.7376	2.7625	2.7934	2.8147
*n* ^T^_2_ × 10^−9^	2.6355	2.6547	2.7089	3.0145
*n* ^F^_2_ × 10^−10^	4.0345	4.1983	4.3781	4.7365

The value of *E*_g_ of the as-prepared film GeTeSe_0.5_ was 1.18 eV,^17^ which initially increased upon the doping of yttrium but then decreased as the Y doping increased. This can be explained by the Mott and Davis model.^33^ After doping, structural transformation takes place and the density of localized states increases in the gap, which decreases the bandgap.^34,35^ In another way, by the chemical bond approach, the formation of greater bond energy, such as for Y–Te (339 kcal mol^−1^) and Y–Se (435 kcal mol^−1^), was less than for Ge–Te (396.7 kcal mol^−1^) and Ge–Se (484.7 kcal mol^−1^). The temperature dependence of the bandgap in semiconductors is often associated with an increase in interatomic separation. This occurs because the magnitude of atomic vibration increases with a rise in thermal energy, leading to enhanced electron–lattice interactions. The P parameter indicates the degree of structural disorder for semiconducting materials. The *P* value was calculated from the slope of the graph shown in Fig. 6. The increasing value of *P* shows the reduction in the structural disorder.

Defects and impurities within a film can lead to a weak absorption region characterized by an exponential variation of ‘α’ with *hv*. This absorption edge signifies the presence of localized states within the gap. Band tailing arises from random variations in internal fields, leading to structural irregularities. The energy linked with this region is referred to as the Urbach energy (*E*_U_), which is calculated using Urbach's relation.4
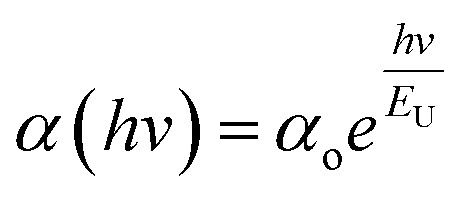
where *α*_o_ represents the absorption coefficient value at the bandgap value. The *E*_U_ value (as presented in Table 2) is calculated as the reciprocal of the slope of the linear fit between the ln(*α*/*α*_o_) and h*v* graph. *E*_U_ reflects the width of the band tails in localized states, indicating the degree of disorder present in the semiconductor. The alteration in *E*_U_ value is related to the density of defect states in the bandgap region.^36,37^ Here, the variations of *E*_g_ and *E*_U_ with the composition are depicted in Fig. 6(b).

The broadening observed in the absorption edge is characterized by the steepness parameter (*σ*), attributed to excitation resulting from electron–phonon or excitation–phonon interactions. This parameter is determined by the relation *σ* = *KT*/*E*_U_, where *T* represents the temperature, *E*_U_ denotes the Urbach energy, and *K* is the Boltzmann constant. The attained *σ* values are provided in Table 2, demonstrating the increase in *σ* with the increase in Y content. This observation confirmed the widening of the optical bandgap due to the broadening of the gap.

The strength of interaction between electrons and phonons is denoted by *S*_e–p_. Information regarding lattice expansion and the increment in lattice constants can be attained from *S*_e–p_. An increase in the *S*_e–p_ value suggests an expansion in the lattice dimensions, and *vice versa*. *S*_e–p_ is calculated using the formula *S*_e–p_ = 2/3*σ*, as illustrated in Table 2. Considering that an increase in lattice dimensions leads to a decrement in the bandgap energy, there was a decrease in the *S*_e–p_ value with the Y content. The decreased *S*_e–p_ values correlate well with the higher *E*_g_ value due to their inverse relationship. Due to the unique optical characteristics of Ge–Te–Se–Y thin films, they are highly regarded for various optoelectronic applications and devices, including photonic circuits, photovoltaics, signal processing, solar cells, photolithography, optical fibers, and optical recorders.^31^

#### 3.3.3. Refractive index and dispersion parameters

The refractive index (*n*) serves as a crucial indicator of light dispersion and is particularly valuable for understanding nonlinear optical phenomena. It plays a significant role in achieving strong optical field confinement, enabling small waveguide bend radii and increased optical intensities. The refractive index offers insightful information concerning both the linear and nonlinear characteristics of a sample. To calculate the refractive index (*n*) from transmission data, the below relation is employed:5
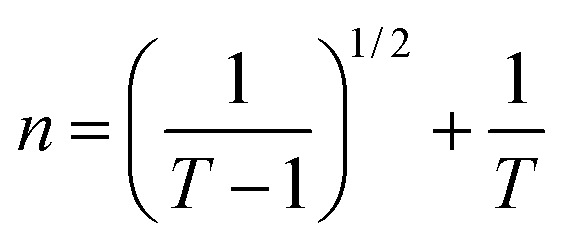


**Fig. 7:**
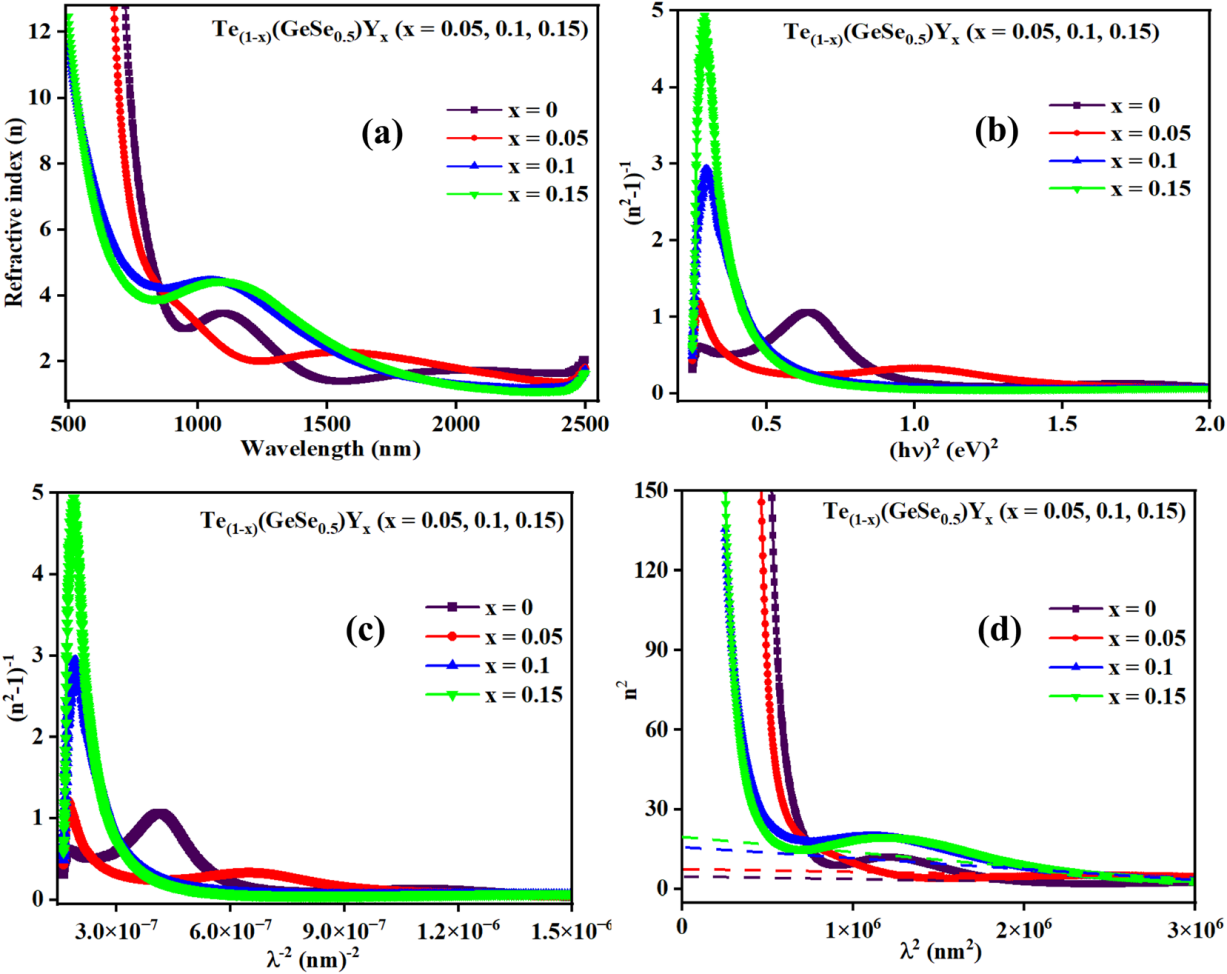
(a) Variation in ‘*n*’, (b) (*n*^2^ − 1)^−1^*versus* (*hv*)^2^ plot, (c) (*n*^2^ − 1)^−1^*versus* (*λ*)^−2^ plot, and (d) *n*^2^*versus* (*λ*)^2^ plot for the Te_(1−*x*)_(GeSe_0.5_)Y_*x*_ thin films.

Fig. 7(a) displays the refractive index variation with wavelength, displaying the normal dispersion nature of the film. The value of ‘*n*’ increased with the doping of Y, with a simultaneous increment in *E*_g_. The value of ‘*n*’ was large at higher wavelength values, whereas it decreased at lower wavelengths. Absorption tends to be higher at higher frequencies, where the speed of light decreases, leading to an increase in the refractive index (*n*). This higher value of ‘n' facilitates greater optical field confinement, enabling switching in phase-change materials. Consequently, the optical intensities are enhanced, rendering the material more efficient and suitable for nonlinear interactions.^21,38^ The variation in refractive index is correlated with changes in the bandgap, as per Moss's rule, where *E*_g_*n*^4^ remains approximately constant.^30,31^

The dispersion parameters, particularly the single oscillator energy (*E*_o_) and dispersion energy (*E*_d_), are crucial for the design and functioning of spectral dispersion and optical communication devices. Two important parameters for optical devices are the single oscillator (*E*_o_) and dispersion energy (*E*_d_). *E*_o_ represents the single oscillator energy, which provides information about the band structure of a material, while *E*_d_ denotes the dispersion energy, which signifies the average interband optical transition strength. *E*_d_ is independent of *E*_o_ as it depends on the dielectric loss and on factors such as the structural parameters of a material. The Wemple–DiDomenico model relates *E*_o_, *E*_d_, and the energy of the incident photons (*hv*). Structural parameters that influence *E*_d_ include the coordination number (*N*_c_), ionicity (*β* = 0.37 eV), effective number of valence electrons per anion (*N*_e_), and anion valency (*Z*_a_) of a material and these are related by *E*_d_ = *βN*_c_*Z*_a_*N*_e_ (eV). *E*_d_ is crucial for understanding the structural disorder and physical characteristics of a material. A decrease in *E*_d_ indicates a reduction in structural disorder, implying improved material properties.

The single effective oscillator model offers a comprehensive physical explanation of the measured parameters. This model is expressed by the following equation:^30,31^6
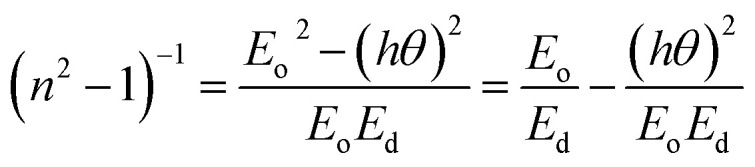
where *E*_o_ and *E*_d_ were calculated from the plot between (*n*^2^ − 1)^−1^*versus* (*hv*)^2^, displayed in Fig. 7(b). These values were calculated by the slope and intercept of the graph, as displayed in Table 2. It was observed that there was an increment in *E*_o_ while *E*_d_ decreased. The static refractive index (*n*_o_) and lattice dielectric constant (*ε*_∞_) were evaluated by using the following relation:^17^7
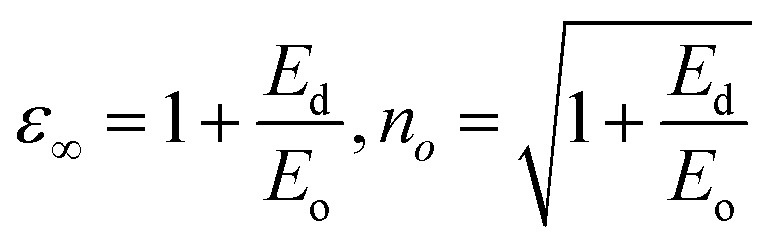


The quantity *f* = *E*_o_*E*_d_ specifies the oscillator strength of a material, which was found to increase with the composition. *ε*_∞_ was noticed to decrease with the yttrium doping. The two factors of the spectra first-order (*M*_−1_) and third-order (*M*_−3_) were calculated by *E*_o_ and *E*_d_ by the below relations:^30,31^

8 *M*_−1_ = *E*_d_/*E*_o_ and *M*_−3_ = *M*_−1_/*E*^2^_o_

The values of both *M*_−1_ and *M*_−3_ both decreased with the yttrium composition, as displayed in Table 1. In the lower frequency regime, the ‘n’ value follows the Sellmeier's dispersion model^17,31^ given below:9
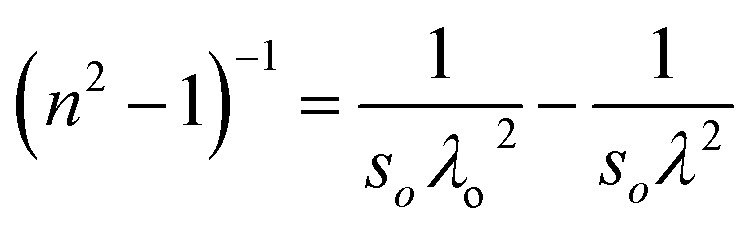


The value of *s*_o_ (strength of oscillator) and *λ*_o_ (wavelength of oscillator) can be determined by a straight-line fitting of the graph between (*n*^2^ − 1)^−1^ and (*λ*)^−2^ (shown in Fig. 7(c)). Here, the values of *s*_o_ and *λ*_o_ decreased with Y addition, as displayed in Table 2*λ*_o_ is inversely related to *E*_o_, and *E*_o_ is directly proportional to *E*_g_, which results in *λ*_o_ ∝ 1/*E*_g_.

#### 3.3.4. High-frequency dielectric parameters

The ‘*ε*_L_’ and carrier concentration per effective mass (*N*/m*) were determined by analyzing the relationship between ‘*n*’ and ‘*λ*’ with the utilization of the following equation:^39–41^10
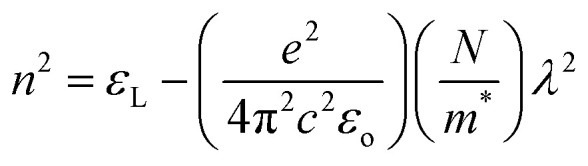


The plot depicted in Fig. 7(d), in correlation, facilitates the assessment of *N*/m* and *ε*_L_, allowing deducing these values from the intercept and slope, respectively. The change in dipole orientation within the chalcogenide films resulted in variations in *ε*_L_. Despite the close proximity of both the *ε*_L_ and *ε*_∞_ values, the decrease observed with the composition could be attributed to the increased polarization. The lower *ε*_∞_ value compared to *ε*_L_ was attributed to the contribution of free charge carriers to the polarization process, consistent with findings from previous studies.^42,43^

The plasma frequency (*ω*_P_) is calculated by using the Drude relation:^17,44^11
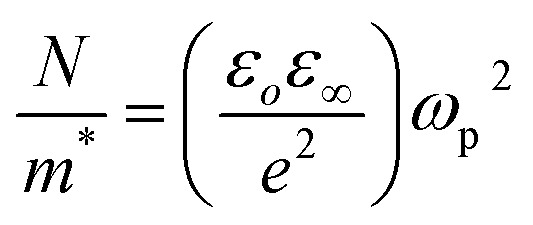
or12
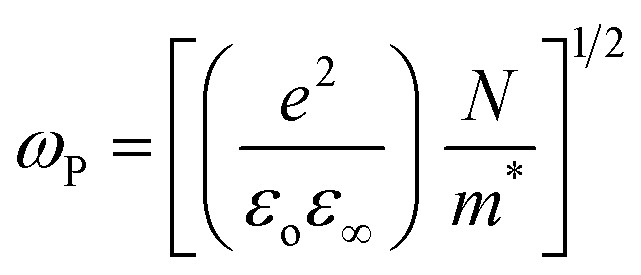
Here, the calculated values of *ω*_P_ are depicted in Table 2. At the plasma frequency, the peak oscillation of free charge carriers occurs. Apart from the plasma frequency, no other resonance was present, and the material's radiation propagated within the dielectric medium, with reflection occurring at lower frequencies.

The dielectric constant is an inherent property of a semiconductor that defines its behavior when subjected to an applied electric field. It comprises both real (*ε*_r_) and imaginary (*ε*_i_) parts, denoted as ε* (=*ε*_r_ + i*ε*_i_). Furthermore, in terms of ‘*n*’ and ‘*k*’, it can be represented as ε* = (*n* + i*k*)^2^. The dielectric loss tangent (tan *δ*) is derived from *ε*_r_ and *ε*_i_, where *ε*_r_ provides insights into the attenuation of incident energy during propagation within the films and is also related to the density of states (DOS) within the forbidden gap of a semiconductor material, while *ε*_i_ accounts for energy loss, referred to as the damping factor. This factor quantifies the energy dissipation within the material as light waves pass through it. The ratio of *ε*_i_/*ε*_r_ assesses the loss factor, and these parameters are determined using the equation below :^17,45^

13 *ε*_i_ = 2*nk* and *ε*_r_ = *n*^2^ − *k*^2^

**Fig. 8:**
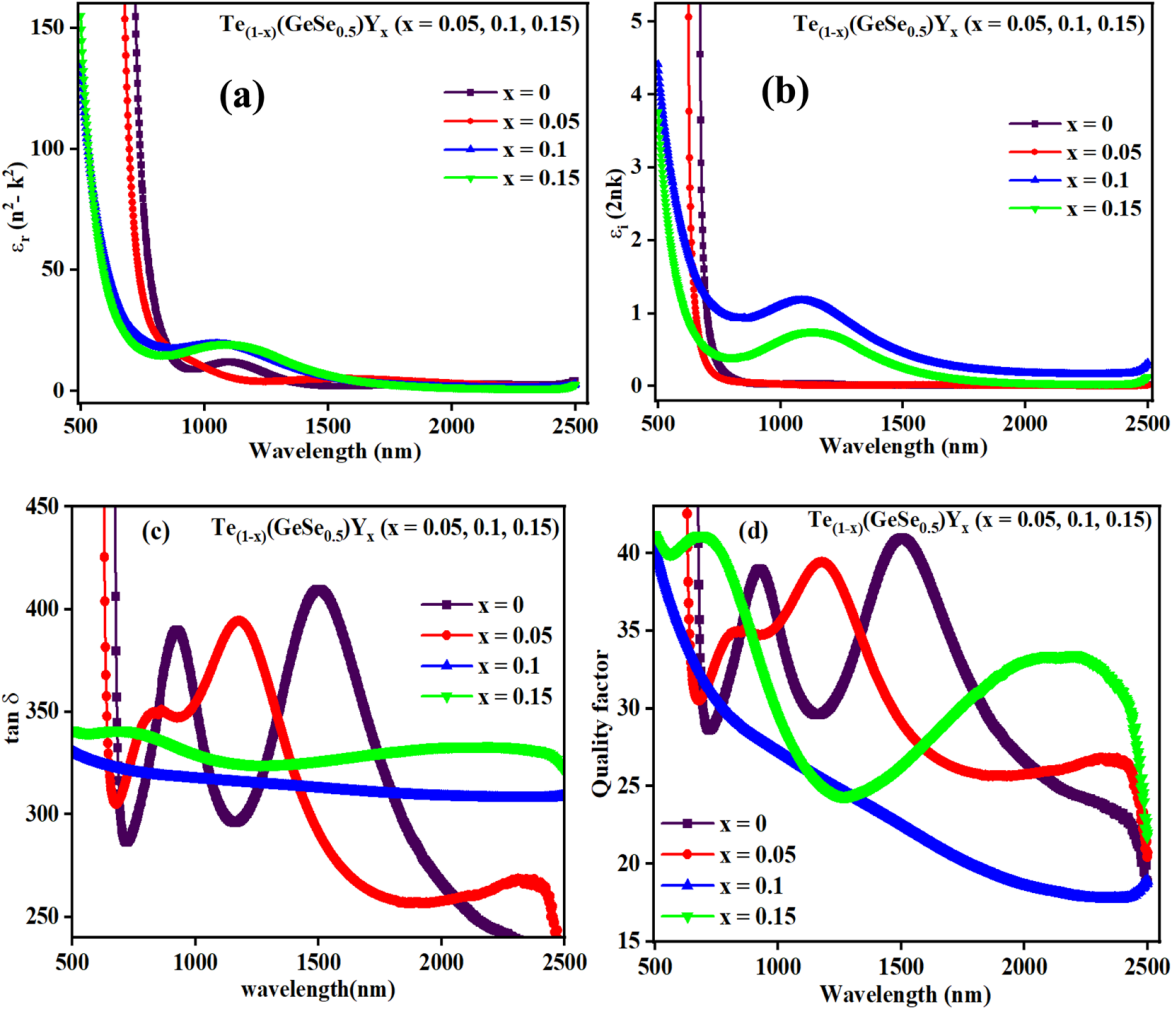
Plots of (a) *ε*_r_, (b) *ε*_i_, (c) tan(*δ*), (d) quality factor *versus* wavelength for the Te_(1−*x*)_(GeSe_0.5_)Y_*x*_ thin films.

Fig. 8(a) and (b) depicts the variation in *ε*_r_ and *ε*_i_ with wavelength, where it can be seen that both decreased with wavelength. The relatively higher value of *ε*_r_ compared to *ε*_i_ resulted from variations in ‘*n*’ and ‘*k*’. The dielectric loss factor quantifies the energy absorbed by the material as electromagnetic waves propagate through it. This parameter also illustrates the phase difference in energy loss at a fixed frequency (refer to Fig. 8(c)). Here, the value of ‘tan *δ*′ was higher at higher photon energy. The change in the nature of tan(*δ*) resembles that of the ‘*k*’ value, indicating the predominant influence of dielectric loss over optical absorption in this electromagnetic regime. The quality of oscillation, known as the quality factor (Q), is the reciprocal of tan(*δ*), *i.e.*, *Q* = *ε*_r_/*ε*_i_. The *Q* factor was observed to decrease with the wavelength for the studied films, as depicted in Fig. 8(d).

When electrons move within a sample, they lose energy due to plasma oscillations induced in a sea of conduction band electrons. This energy loss is closely tied to the optical properties of the material. Energy loss parameters are crucial for understanding damping effects on the surface or within films. These functions help measure the average free path of inelastic electrons as they traverse through the sample. Therefore, it is vital to compute the volume energy losses (VELF) and surface energy loss functions (SELF). VELF and SELF are determined using *ε*_r_ and *ε*_i_ as follows:^46^14
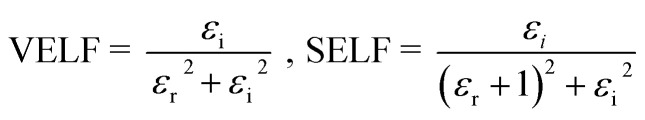


**Fig. 9:**
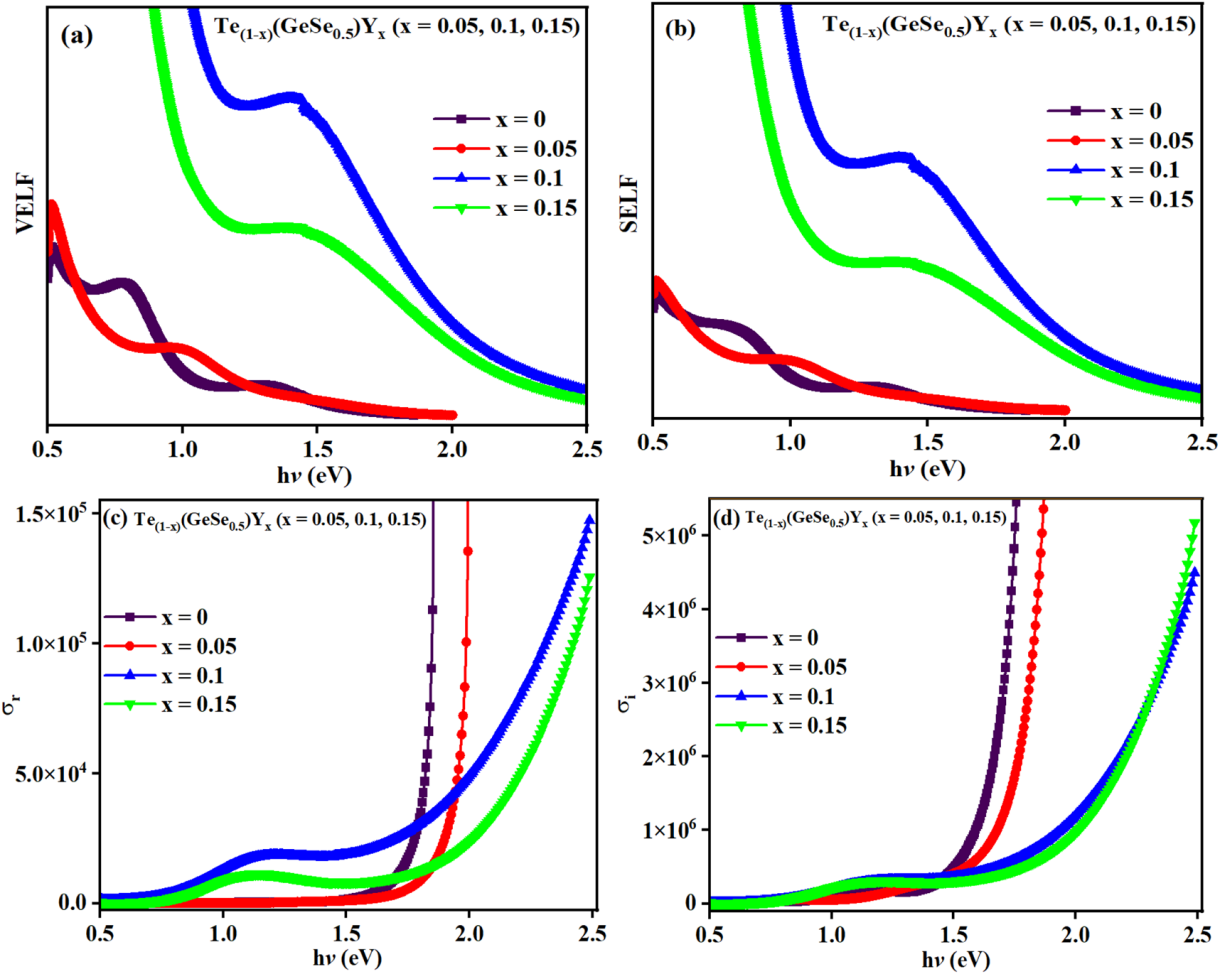
(a) Variation in VELF, (b) SELF, (c) *σ*_r_, and (d) *σ*_i_ with *hv* for the studied films.

Both VELF and SELF characterize the absorption of energy in a material, indicating losses and are correlated with a single electron transition in a semiconductor. Fig. 9(a) and (b) display the variations in VELF and SELF with ‘*hv*’, illustrating that VELF and SELF decreased with *hv*.

#### 3.3.5. Optical electronegativity (*η*_opt_) and optical conductivity

The optical response of a sample is assessed through its optical conductivity (*σ*) value, providing insight into the extent of light propagation within the medium across specific wavelength ranges. The investigation of the complex ‘*σ*’ of films is crucial due to their functional applications. The ‘*σ*’ value relies on *ε*_r_ and *ε*_i_, reflecting the electronic state density within the material's bandgap. The complex optical conductivity, *σ*_opt_* = *σ*_r_(*ω*) + i*σ*_i_(*ω*), has both real and imaginary parts, where *σ*_i_(*ω*) = *ωε*_o_*ε*_r_ and *σ*_r_(*ω*) = *ωε*_o_*ε*_i_, where *ω* denotes the angular frequency. Fig. 9(c) and (d) depict the variation in real and imaginary optical conductivities with photon energy. Both exhibited a rapid rise as energy increases. This surge in optical conductivities is attributed to increased absorption coefficients at higher energies or lower wavelengths, a trend observed in prior studies as well.

The electrical susceptibility (*χ*_c_), *i.e.*, the degree of polarization, is calculated by:15
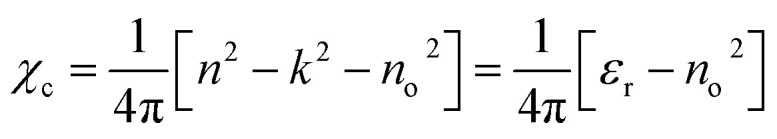


**Fig. 10:**
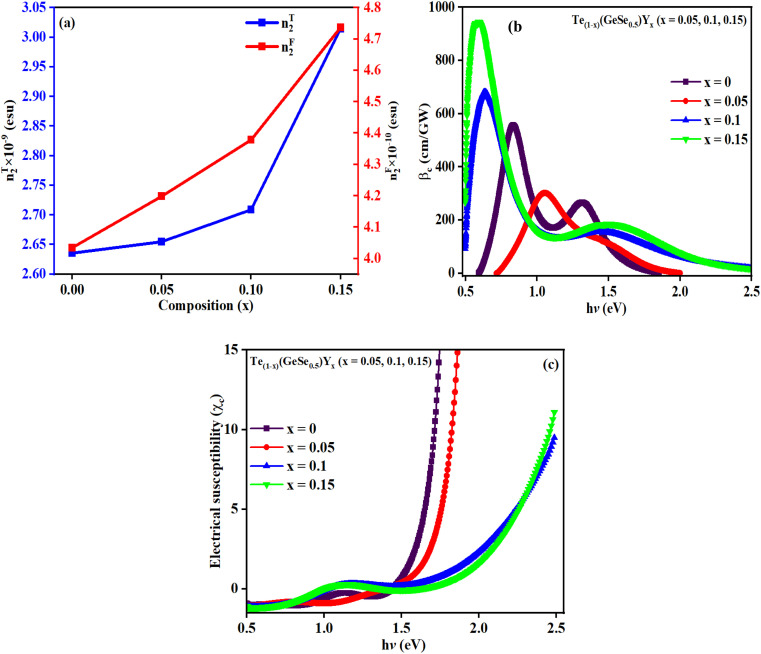
(a) *n*^T^_2_ and *n*^F^_2_ variation with the composition, (b) nonlinear absorption the coefficient *β*_c_, (c) electrical susceptibility (*χ*_c_) variation with *hv* for the studied thin films.

The value of *χ*_c_ is displayed in Fig. 10(c), which displays an increasing trend with *hv*. The presence of lone pairs is associated with polarization by optical electronegativity (*η*_opt_). The value of *η*_opt_ in terms of *n*_o_ is expressed as follows: *η*_opt_ = (*C*/*n*_o_)^1/4^, where *C* = 25.54. The calculated *η*_opt_ values exhibited an increase with the composition for the studied films, as indicated in Table 2. Since *η*_opt_ is correlated with the refractive index, optical electronegativity also influences the nonlinear parameters.

#### 3.3.6. Nonlinear optical properties

Nonlinearity in films arises when a high-intensity electromagnetic wave interacts with them, governed by the Kerr effect: Δ*n*_o_ = *n*_2_ × *I*, where *I* represents the optical intensity and *n*_2_ denotes the nonlinear refractive index. The nonlinearity originates from the interactions of electronic polarization, which influence the bond length of the material. Understanding the nonlinear optical properties of a material is crucial for determining the propagation characteristics of light through it. The polarization *P* can be expressed as below:

16a *P*(*t*) = ε_o_[*χ*^1^*E*(*t*) + *χ*^2^*E*(*t*)^2^ + *χ*^3^*E*(*t*)^3^ + …]

where *E*(*t*) signifies the electric-field strength, and *ε*_o_ denotes the permittivity in free space. The electrical susceptibility comprises both linear susceptibility (*χ*^1^) and nonlinear susceptibilities (*χ*^2^, *χ*^3^). Materials exhibiting inversion symmetry have even-order terms set to zero for nonlinear susceptibility, *i.e.*, *χ*^2^ = 0. The linear susceptibility *χ*^1^ is determined by the given equation:16b
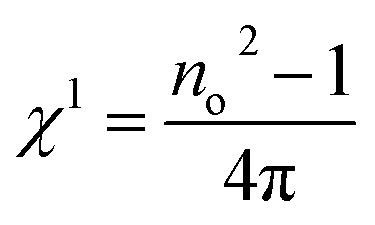
where ‘*n*_o_’ defines the linear refractive index. Here, the values of *χ*^1^ are displayed in Table 2, indicating a decrease with the composition of the studied films. Miller's generalized rule was employed to derive the third-order nonlinear optical susceptibility (*χ*^3^):

16c *χ*^3^ = *A*(*χ*^(1)^)^4^

where *A* equals 1.7 × 10^−10^ esu. The values of *χ*^3^ for the various studied films are detailed in Table 2, indicating a decrease with Y doping. The presence of polarized atoms possessing lone pairs (such as chalcogens) influences the *χ*^3^ value, but heavy atoms with easily polarized electron clouds (*e.g.*, In) are even more impactful. Here, the observed monotonic decrease in nonlinear susceptibility was attributed to changes in the material structure resulting from the compositional change.

The nonlinear refractive index is determined from *χ*^3^ using the Tichy and Ticha formula:^17,30,31^16d
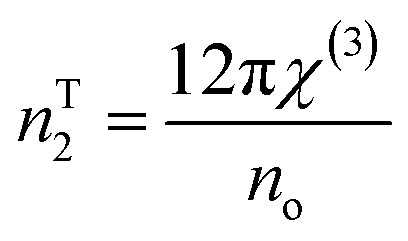
where the value of *n*^T^_2_ decreased with the wavelength of the studied films, as indicated in Table 2. Fornier and Snitzer established a correlation between *n*^F^_2_ and the linear refractive index of the material utilizing WDD parameters, as given below:16e
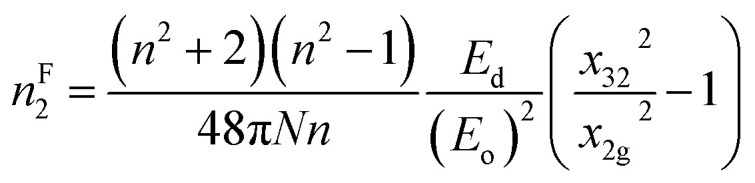
where ‘*n*’ represents the refractive index, ‘*N*’ signifies the density of polarizable constituents, and the subscripts g represent the ground state (g) and excited states (2 and 3), respectively. When considering the three-level system within this model, the expression ((*x*_32_2/*x*_2g2_) − 1) = 1 is used, with ‘*n*’ representing the static refractive index, *n*_o_. Therefore, the aforementioned equation can be expressed as follows:^30,31^16f
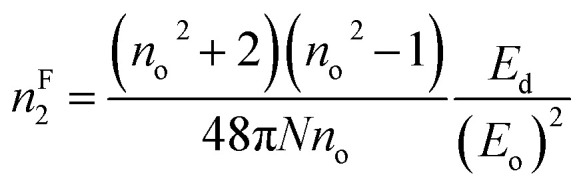


The estimated *n*^F^_2_ using the Fornier and Snitzer models is displayed in Table 2. Compared to *n*^T^_2_, *n*^F^_2_ exhibited a lower value. The comparative graph depicted in Fig. 10(a) illustrates that both *n*^F^_2_ and *n*^T^_2_ followed a similar trend to the linear refractive index, showing an overall decrease with the rise in wavelength. The number of homopolar bonds decreased with the increment in heteropolar ones, thereby reducing the defects in the band structure. Consequently, the reduction in defect density and variety accounted for the decrease in nonlinearity with the compositional variation. The decrease in *χ*^3^ and *n*_2_ by Y content is advantageous for solid-state lasers and UV nonlinear materials.^30,31^

Two-photon absorption (TPA) is a fundamental mechanism that elucidates the process of induced absorption within materials. This phenomenon occurs exclusively when the energy of incident light falls within the range of *E*_g_/2 < *hv* < *E*_g_. The TPA mechanism is characterized by the nonlinear absorption coefficient (*β*_c_), which can be determined using a straightforward empirical relation.^17,31^16g
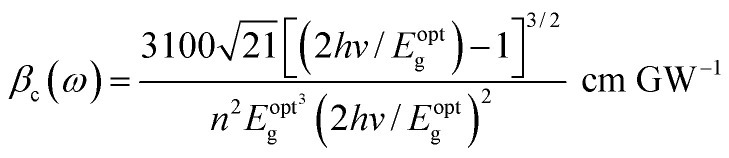
where the magnitude of the *β*_c_ value was higher at lower energy levels and subsequently decreased. Fig. 10(b) illustrates a similar pattern for *β*_c_ as observed with the nonlinear parameters, indicating an increase with wavelength.

## 4. Conclusion

The impact of Y concentration on various factors, including the structural and optical properties of Te_(1−*x*)_(GeSe_0.5_)Y_*x*_ (*x* = 0, 0.05, 0.1, 0.15) chalcogenide thin films, was investigated. The energy bandgap (*E*_g_) values were determined in the strong absorption region using the Tauc extrapolation method. The optical absorption outcomes of the selected system favor allowed indirect transitions. The evaluated optical bandgap was influenced by Y incorporation due to the higher binding energy of Te–Y compared to Te–Te binding energy. Additionally, the decreasing electronegativity contributed to the decrease in bandgap with Y concentration. The absorption coefficient followed the Urbach relation over a wide range of wavelengths, with calculated values of *α* indicating suitability for optical data-storage applications due to the high absorption coefficient. The decreasing *k* value with Y concentration indicated a reduced loss (*ε*_i_ = 2*nk*) due to increased energy absorption by the charge carriers. The nonlinear refractive index was evaluated using various methods, revealing the third-order nonlinear optical susceptibility *χ*^(3)^ (∼10^−10^) and nonlinear refractive index *n*_2_ (∼10^−9^) increase with Y concentration. The results attained for Te_(1−*x*)_(GeSe_0.5_)Y_*x*_ (*x* = 0, 0.05, 0.1, 0.15) thin films show they hold potential for various applications, including optical memory devices.

## Data availability

Data will be made available on request.

## Conflicts of interest

The authors declare no competing interests.
